# HART-c: Prehospital triage via hospital consultation—the future lies in the ambulance

**DOI:** 10.1007/s12471-023-01783-2

**Published:** 2023-04-24

**Authors:** Arnoud W. J. van ’t Hof, Rudolf Tolsma

**Affiliations:** 1 Department of Cardiology, MUMC, Maastricht, The Netherlands; 2 Department of Cardiology, Zuyderland MC, Heerlen, The Netherlands; 3 Cardiovascular Research Institute Maastricht (CARIM), Maastricht, The Netherlands; 4 Emergency Medical Service, Ambulance IJsselland, Zwolle, The Netherlands

The future of improved care in treating the right patient at the right place lies in the ambulance. In recent years, more and more initiatives have been developed showing that, besides general practitioners, ambulance personnel have a prominent role in prehospital triage and referral decision-making. They are highly trained, work strictly according to a national protocol and often participate in complex randomised controlled trials.

This situation in the Netherlands is unique in the world; only a few countries have comparable emergency medical systems [1, 2]. Many innovations in the prehospital care of acute cardiac patients were co-initiated in the Netherlands. For example, the prehospital diagnosis of acute ST-elevation myocardial infarction (STEMI), which paved the way to the prehospital treatment with thrombolysis and later to immediate referral and transfer to the nearest intervention centre to perform primary percutaneous coronary intervention (PCI). Since early this century, patients without ST elevation on the diagnostic ECG are also part of scientific studies aiming to improve logistics, triage and treatment of patients with (suspected) acute myocardial infarction or other acute cardiac pathology.

De Koning et al. describe the results of the HART‑c project, another example of such a new development [3]. The study showed that a prehospital triage method in patients with cardiac symptoms, combining prehospital ambulance data with expert consultation of a cardiologist in the hospital, resulted in more patients who could safely stay at home. This number increased from 77 (5.9%) to 181 (11.8%). Only one patient (< 1%) who stayed at home developed an acute coronary syndrome (ACS) within 30 days after the ambulance visit. In addition to a role in decision-making, the consulted cardiologist also had insight into the admission capacity of multiple hospitals in the region, allowing a decision to be made on which hospital the patient could best be referred to. This led to a significant decrease in the number of inter-hospital transfers (173/1355 in the intervention group vs. 206/1299 in the control group. Relative risk 0.81 (95% CI 0.67–0.97, *p* = 0.023)).

Previous studies, such as FamouS Triage, have shown that keeping low-risk patients at home after prehospital risk stratification using the HEART score, is feasible and non-inferior to transferring all patients to the hospital [4]. In addition, it was shown that a prehospital troponin measurement on top of such a risk stratification instrument can improve the selection of patients. It is expected that new high-sensitive point-of-care troponin devices can even further improve the safety of prehospital stratification [5–7]. The recent ARTICA trial showed that this is also cost-effective [8]. This way, up to 20–30% of patients who call the emergency telephone number for the Netherlands (112) because of chest pain can safely stay at home. These patients often have non-cardiac chest pain or other low-risk conditions that do not require further evaluation at the emergency department.

The HART‑c project brings a new development to this armamentarium: the need for exchange of data between the ambulance and the hospital combined with consultation with a cardiac specialist. This exchange of information resulted in pre-informed arrival of the patient at the right hospital, less inter-hospital transfers and high-rated satisfaction scores for both the patient, ambulance and hospital personnel. The development of a special data exchange platform is crucial in this regard but not easy: patient data is prone to leaks and the challenges of exchanging patient data within the General Data Protection Regulation are well known. Fortunately, more and more examples show that secure data exchange between ambulance and hospital is possible, and associated with improved patient outcome and less costs [9, 10].

There is a great need to rapidly implement successful projects such as HART‑c, FamouS Triage, ARTICA and URGENT in the Netherlands. All are examples of diagnosing and treating patients in the right place. Hospitals should only diagnose and treat intermediate and high-risk patients. Nowadays, low-risk patients are still consuming hospital expenses when referred to the hospital. Hospitals are experts at ruling out diseases and work more defensively: often additional CT scans, laboratory tests and even cardiac catheterisations are performed on these low-risk patients, without additional value but at a cost of about 7500 euro per patient. It is estimated that 30–50 million euro could be saved nationwide when these unnecessary hospital admissions are prevented [8, 11].

The best way to go is to combine the results of the above-mentioned projects and other similar studies: prehospital risk stratification with the HEART score, including high-sensitive point-of-care cardiac troponin measurements together with the support of consultation with an medical specialist at the hospital who also has access to a data information platform.

This strategy will also be applicable in primary care where general practitioners see patients with chest pain in their own practice or out-of-hours clinic. The HART‑c project is a plea for more collaboration in the chain of acute care, not only at the patient level, but also with regard to resolve barriers in data exchange.

## References

[1] Backus BE, Ter Avest E, Gerretsen BM (2020). Organization of prehospital care in the Netherlands: a perspective article. Eur J Emerg Med.

[2] Backus BE, Tolsma RT, Boogers MJ (2020). The new era of chest pain evaluation in the Netherlands. Eur J Emerg Med.

[3] de Koning E, Beeres S, Bos J (2023). Improved prehospital triage for acute cardiac care: results from HART-c, a multicentre prospective study. Neth Heart J.

[4] Tolsma RT, Fokkert MJ, van Dongen DN (2022). Referral decisions based on a pre-hospital HEART score in suspected non-ST-elevation acute coronary syndrome: final results of the FamouS Triage study. Eur Heart J Acute Cardiovasc Care.

[5] van Dongen DN, Fokkert MJ, Tolsma RT (2018). Value of prehospital Troponin assessment in suspected non-ST-elevation acute coronary syndrome. Am J Cardiol.

[6] Bruinen AL, Frenk LDS, de Theije F (2022). Point-of-care high-sensitivity troponin-I analysis in capillary blood for acute coronary syndrome diagnostics. Clin Chem Lab Med.

[7] Koper LH, Frenk LDS, Meeder JG (2022). URGENT 1.5: diagnostic accuracy of the modified HEART score, with fingerstick point-of-care troponin testing, in ruling out acute coronary syndrome. Neth Heart J.

[8] Camaro C, Aarts GWA, Adang EMM (2023). Rule-out of non-ST-segment elevation acute coronary syndrome by a single, pre-hospital troponin measurement: a randomized trial. Eur Heart J.

[9] B.V. I. AZN Connect, wanneer elke seconde telt. 2023. https://www.aznconnect.nl/ (Created 02.2023).

[10] Minderhout RN, Vos HMM, van Grunsven PM (2021). The value of merging medical data from ambulance services and general practice cooperatives using triple aim outcomes. Int J Integr Care.

[11] van Dongen DN, Ottervanger JP, Tolsma R (2019). In-Hospital Healthcare Utilization, Outcomes, and Costs in Pre-Hospital-Adjudicated Low-Risk Chest-Pain Patients. Appl Health Econ Health Policy.

